# Retraction Notice: Transition in Dental Treatment Utilization in Jammu And Kashmir, India - A 10 Year Retrospective Study

**DOI:** 10.3126/nje.v7i3.19009

**Published:** 2017-09-30

**Authors:** Aasim Farooq Shah, Manu Batra, A. Ishrat

**Affiliations:** 1 Registrar, Department of Public Health Dentistry, Government Dental College & Hospital Shreen Bagh, Srinagar, Jammu and Kashmir, India. 190010.; 2 Assistant Professor, Department of Community and Preventive Dentistry, UCMS College of Dental Surgery Bhairahawa, Nepal.; 3 Dental Surgeon [School Health Program], J&K Government Health Services. Srinagar, Jammu and Kashmir India 190001.

RETRACTED ARTICLE:It was brought to the editorial notice, that this article has fabricated data. This was confirmed and Authors were communicated; they acknowledged and agreed that this has been done by mistake. To avoid misleading the scientific community the article was retracted on 30th September 2017 (DOI:http://dx.doi.org/10.3126/nje.v6i4.17257).

This retracts the article " Transition in Dental Treatment Utilization in Jammu And Kashmir, India - A 10 Year Retrospective Study" in volume 6 on page 631.

